# Stories of Backlash in Interviews With Survivors of Intimate Partner Violence Against Women in Sweden

**DOI:** 10.1177/10778012221088312

**Published:** 2022-07-11

**Authors:** Maria Wemrell

**Affiliations:** 1 Unit for Social Epidemiology, Medical Faculty, 5193 Lund University, Lund, Sweden; 2 Department of Gender Studies, Faculty of Social Sciences, 5193 Lund University, Lund, Sweden

**Keywords:** intimate partner violence against women, backlash, gender equality, Nordic Paradox, Sweden

## Abstract

Despite high gender equality ratings, Sweden shows a high prevalence of intimate partner violence against women (IPVAW). Suggested factors underlying this apparent paradox include backlash effects against women's empowerment. This study explores stories of backlash in interviews with 23 IPVAW survivors in Sweden. Thematic analysis identified categories of narrative segments referring to phenomena provoking violence; the victims’ *resources*, *agency*, breaking with *gender norms* and *resistance*, and the *partner's feelings of subordination*, while case-centered narrative analysis pointed to divergences between how these categories appear in the stories. The study underscores the complexity of links between gender (in)equality and IPVAW in Sweden.

## Introduction

Intimate partner violence against women (IPVAW) is a global human rights and public health issue, to which one-third of women are estimated to be exposed during their lives (WHO, 2019), that is typically perceived as being associated with gender inequality (e.g., Council of Europe, 2011). Although Sweden has been rated as one of the most gender-equal countries in the EU and the world (EIGE, 2019; World Economic Forum, 2019), its IPVAW prevalence appears to be comparatively high (FRA, 2014). This seemingly contradictory co-existence of high levels of gender equality and of IPVAW found in Sweden and other Nordic countries (FRA, 2014) has been termed the Nordic Paradox (Gracia & Merlo, 2016). One factor suggested to possibly underlie this paradox (Gracia & Merlo, 2016) is backlash effects against women's empowerment. While this notion has found some support in quantitative studies (Bjelland, 2014; Ericsson, 2020), qualitative research on experiences of backlash dynamics in the context of IPVAW is sparse. This article aims to explore stories of backlash in interviews with survivors of IPVAW in Sweden, through thematic and case-centered narrative analysis.

### IPVAW and Gender Equality

IPVAW can be defined as physically, sexually, psychologically, or economically coercive acts against women by their current or former partners (UN, 2006), including violence online and via information and communications technology (OHCHR, 2018), without their consent. While intimate partner violence (IPV) occurs in LGBTQI relationships, and men are exposed, a large share of victims are (cis- or transgender) women (Heise & Garcia-Moreno, 2002).

Different explanatory models for IPVAW place emphasis on various causal factors, such as socioeconomic deprivation (Xie et al., 2012) or more individually or relationally oriented aspects including mental health issues (Yu et al., 2019), substance abuse (Kantor & Straus, 1987), lack of impulse control (Dutton, 1997), or behavioral patterns transmitted between generations (Stith et al., 2000). In line with the ecological model recommended by the WHO for IPVAW research (Heise, 1998), which integrates individual, relational, and structural levels, the causes of IPVAW are generally seen as complex (e.g., Hydén et al., 2016).

That said, IPVAW is commonly perceived as being tied to gender inequality (e.g., Council of Europe, 2011). The European Institute for Gender Equality (EIGE, 2017) notes that IPVAW can be seen as both a driver and an effect of gender inequality, and as a form of gender inequality in itself. In Sweden, the link between IPVAW and gender inequality is well established in political discourse and policy (e.g., Prop, 2005/06: 155; Skr, 2016/17: 10). Correspondingly, a large share of research on IPVAW, and on violence against women (VAW), is concerned with its association with gender inequality. Such research is not, however, entirely clear about the nature of that link, as two hypotheses—the amelioration and the backlash hypotheses—exist side by side (e.g., Roberts, 2011; Vieraitis et al., 2015).

The amelioration hypothesis posits that gender equality mitigates against, and should therefore be associated with a lower prevalence of, IPVAW. The concept of backlash, in turn, links violence to retaliation effects due to redefined power relations involving men's (perceived) loss of control, in the context of advances towards gender equality (e.g., Whaley & Messner, 2002). While these hypotheses point in opposite directions, positing that gender equality will cause VAW to decrease or increase, respectively, they have both found support in quantitative empirical research (e.g., Roberts, 2011; Vieraitis et al., 2015). Thus, some studies point to higher levels of gender inequality being associated with lower levels of violence (e.g., Aizer, 2010; Titterington, 2006), while others support the backlash hypothesis by pointing to a reverse association (e.g., Franklin & Menaker, 2014; Gillespie & Reckdenwald, 2017). Conflicting results on this point can even be found in the same studies, depending on, for example, which geographical area (Sabarwal et al., 2014; Whaley & Messner, 2002) or which population group (Pridemore & Freilich, 2005) is in focus. Roberts (2011) concludes that the direction of the relationship between gender equality and VAW cannot be assumed, while Whaley and Messner (2002) observe that it is more complex than is often recognized.

Emphasizing the importance of investigating social conditions under which ameliorative and backlash processes occur, Whaley et al. (2013) suggest that the two operate at different levels of gender equality. Using femicide data from the United States, and invoking the image of an inverted U, they indicate that VAW increases due to backlash processes as gender equality develops from low to intermediate levels, while amelioration processes become more influential where levels of gender equality range from intermediate to high. Similarly, and also affirming the importance of understanding amelioration and backlash dynamics for effective IPVAW interventions, Schuler and Nazneen (2018) associate increased IPVAW due to backlash with incipient empowerment, during which women's enhanced position is viewed, on the community level, as transgressive. Amelioration dynamics and lower levels of IPVAW are linked, in turn, to normative empowerment, where women's rights are more established. Supported by data from Bangladesh, Schuler and Nazneen thereby suggest that as gender equality develops, women's empowerment evolves from being a risk factor for IPVAW to becoming a protective factor. Correspondingly, studies noting differences between geographical regions point to a stronger presence of IPVAW, and thus of backlash, in contexts where gender norms are more conservative (Sabarwal et al., 2014; Whaley & Messner, 2002). Vieraitis et al. (2015) also point to initial gains in gender equality being met by increased femicide rates, followed by a long-term decrease. Thus, based not only on the amelioration hypothesis, but also on studies integrating findings on amelioration and backlash, IPVAW would be expected to be less common in countries with highly rated gender equality.

Nevertheless, in a study of European countries during 1990–2005, Stamatel (2014) finds that nations with less traditional gender roles, as indicated by family dynamics, had higher femicide rates. In a later study, Stamatel (2018) observes a U-shaped relationship between financial equality and femicide, rather than the inverted U noted by Whaley et al. (2013). The femicide rates were high in countries with low financial gender equality, but the decreasing trend associated with increasing gender equality turned into rising rates in countries with higher levels of gender equality. Looking at the separate domains of the EIGE (2015) gender equality index (GEI)—money, knowledge, work, power, time, and health—Stamatel notes that while the data on financial gender equality support the backlash hypothesis, health equality reduced the risk of femicide while the other domains showed no significant correlations. This underscores, again, the complexity of gender inequality and its links with VAW.

As also noted by Stamatel (2018), the observed association between financial gender equality and higher femicide rates is in line with the results of an EU-wide survey (FRA, 2014), which shows a correlation between higher country-level gender equality and higher IPVAW prevalence (Ericsson, 2020). The FRA (2014) data also display a U-shaped correlation, where IPVAW rates are higher among women who have a much lower or, particularly, a higher income than their partner (European Commission, 2017).

### IPVAW in Sweden and the Nordic Paradox

Alongside its neighboring Nordic countries, Sweden has been rated as one of the most gender-equal countries in the EU (EIGE, 2017, 2019) and the world (World Economic Forum, 2019). One of the stated goals of Sweden's national gender equality policy is to eradicate men's violence against women (Prop, 2005/06: 155; Skr, 2016/17: 10), and Sweden has been assigned a position of international leadership in the area of IPVAW law and policy (Corradi & Stöckl, 2014; GREVIO, 2019), although limitations in their implementation have also been pointed out (GREVIO, 2019; Kvinnolobby, 2021). Meanwhile, reported IPVAW rates in Sweden are among the highest in the EU (FRA, 2014), with a share of women indicating exposure to physical or sexual IPVAW (28%) well above the EU average (22%) (FRA, 2014). A high IPVAW rate in Sweden has also been indicated by other studies (e.g., Korkmaz, 2021; NCK, 2014). According to Eurostat (2021), lethal IPVAW is also relatively common in Sweden. In 2018, Sweden's rate (0.44 per 100,000 inhabitants) was higher than the average (0.30) of, and the fourth highest among, the 19 EU member states showing data.

Factors suggested or discussed as potentially explaining this apparent Nordic Paradox (Gracia & Merlo, 2016) include various forms of reporting or measurement biases (Gracia & Merlo, 2016; Humbert et al., 2021; Martín-Fernández et al., 2020; Permanyer & Gomez-Casillas, 2020; Wemrell et al., 2021). Other suggested explanations are features of Nordic societies such as alcohol consumption patterns (Gracia & Merlo, 2016) or individualism (Wemrell et al., 2021), disproportionally high IPVAW rates in some societal groups (Gracia & Merlo, 2016), and unanticipated consequences of relative gender equality including backlash effects (Gracia & Merlo, 2016; Wemrell et al., 2020; Wemrell et al., 2021).

The latter suggestion is obviously congruent with the backlash hypothesis posited and tested in international research. The view of the co-existence of high levels of gender equality and IPVAW as paradoxical is based on the amelioration hypothesis, and the backlash hypothesis does find some support in Nordic studies. Bjelland (2014) found that women with a higher income or education than their partners were more likely to be exposed to IPVAW in Norway, while Ericsson (2020) identified a higher risk in Sweden of hospital admittance due to violence among women with a higher income, although a U-shaped association also pointed to a higher income decreasing the risk among women with low education. Other researchers have tied IPVAW to challenged masculinity norms (Gottzén, 2014) or to men's feelings of powerlessness (Isdal, 2001), and increased conflict levels due to efforts towards gender equality have been indicated (Grönlund & Halleröd, 2008). In a study of discussions among IPVAW professionals in Sweden (Wemrell et al., 2021), of the factors possibly explaining the Nordic Paradox (Gracia & Merlo, 2016), backlash dynamics found the most support.

While such studies suggest that backlash dynamics may indeed be relevant for IPVAW in Sweden, researchers have also repeatedly pointed to a co-existence of explanatory models for IPVAW in Sweden (e.g., Wemrell et al., 2019), where structural perspectives focused on gender and power dynamics have arguably become less important (Holmberg et al., 2015) or tended to fall away in practice (Edin et al., 2008; Holmberg & Bender, 2003; Mattsson, 2011). This has coincided with the direction of a large share of attention towards individual-level factors and particularly vulnerable groups, and with the use of more gender-neutral perspectives (e.g., Hoppstadius, 2018). A relative absence of focus on gender perspectives, noted, for example, in Swedish health-care (Henriksen et al., 2017; Pratt-Eriksson et al., 2014), judicial (Agevall, 2012; Brännvall, 2016), and social service (Hoppstadius, 2018; Mattsson, 2011) organizations, can be associated with an assumption of existing gender equality (Ekström, 2018; Wemrell et al., 2021). Meanwhile, it has been noted that assumptions of gender equality may exacerbate obstacles to reporting and addressing IPVAW in Nordic countries, for example, through stifling discussions of IPVAW (Clarke, 2011), contributing to barriers to leaving violent partners (Enander, 2010b) and being conducive to victim-blaming attitudes (Gottzén & Korkmaz, 2013). This suggests a need for further understanding of how gender, including backlash dynamics, may function in the context of IPVAW in Sweden.

This study explores stories of backlash in interviews with 23 women exposed to IPVAW in Sweden, through narrative analysis.

## Methods and Material

### Participants and Interviews

This interview study was conducted as part of a larger research project centered on the Nordic Paradox (Gracia & Merlo, 2016), and had the aim of exploring women's narratives about their experiences of IPVAW and gender in/equality. Backlash dynamics was a potential area of interest due to being a suggested possible explanation for the paradox (Gracia & Merlo, 2016), but not a direct focus of the study or the interview questions. The interview content related to backlash is presented here, while other aspects of the material are discussed elsewhere.

The 23 participants were recruited through an invitation distributed to women's shelters and municipal support units, posted on Facebook by a women's shelter, and then shared. Some were contacted directly after disclosing experiences of IPVAW in social media or in a professional context, or through being asked by another participant. While a couple of the women had worked professionally in the area of IPVAW, all were recruited on the basis of their personal experiences of exposure. The invitation letter did not distinguish between cis- and transgender women. All the shared stories are about violent heterosexual relationships.

The participants were 21–54 years old. Most had been subjected to IPVAW in one relationship, lasting 1.5–23 years, while a few had been in more than one violent partnership. All but one had separated from their violent partner/s, on average around six years ago. Eleven of the women had children with their perpetrators. Many were working professionals, several in managerial positions, some were students, and two were on sick leave. A majority had or were attaining a university education. In the (main) violent relationship, the incomes of the partners were said to have been fairly equal by eight of the women, while the partner earned more in nine cases and the woman in six. Their educational levels were described as similar by 12 women; the woman was more highly educated in eight cases and the partner in three. Around half described themselves, without being prompted, as being strong or independent.

As the study was grounded in an interest towards the Nordic Paradox and the Swedish context, and due to noted tendencies in Sweden towards locating IPVAW in other countries or cultures, and towards understanding IPVAW perpetrated or suffered by Swedish versus non-Swedish persons as different (Hoppstadius, 2018; Karlsson et al., 2021; Wemrell et al., 2019), the invitation was directed to women self-identifying as Swedish. During the interviews, the women were asked about their self-identified nationality, and that of the violent partner. In 13 cases, both victim and perpetrator were referred to as Swedish. Of the women, 16 identified as Swedish, two had been adopted from another country, and five had some other form of non-Swedish background. Of the violent partners, 16 were referred to as Swedish or Nordic, and six as having a non-Swedish/Nordic background. One woman had been exposed to violence from both Swedish and foreign-born perpetrators.

The women described a spectrum of degrees and types of violence. All referred to physical violence, occurring occasionally, continuously, or cyclically, including being kicked, bitten, beaten with fists, pushed down stairs, or thrown out of a moving car. Nine women had received murder threats, and two mentioned murder attempts. All described psychological violence, including being verbally abused, “brainwashed,” locked in, or stopped from sleeping or eating. Many described sexual violence, economic or material violence, or violence using information technology. Some referred to violence acted out through children or support institutions. Most described the violence as having developed gradually, in some cases not until after several years. Some spoke about violence continuing after the separation, or as still ongoing. All described violence which could be categorized as intimate terrorism (Johnson, 2008), that is, the partner used violence to gain and maintain control, while the victim did not.

The interviews took place at Lund or Linnaeus University, over the phone, or at the participant's home. Of the 23 women, 19 were interviewed by the author and four by Linda Hiltunen, PhD. The interviews lasted 1–2.5 h, and one follow-up interview was held. After giving informed consent to participation, the women were asked initial questions about their life situations. They were then asked to speak about the violent relationship/s: how it (or they) had evolved, what kind of violence they contained, what the women thought was the cause of that violence, and what support she had received. Questions were also asked about aspects of gender (in)equality in the violent relationship(s).

The interview questions were open, in line with Riessman's (2008) recommendation regarding data collection for narrative analysis of opening up topics and letting participants construct answers in ways they find meaningful, and aimed to cohere with Hydén's (2014) “teller-focused interview” approach. The latter, developed in the context of IPVAW research, emphasizes the importance of a relational space where the participant feels safe and in control, and of listening to and supporting the participant's narrative. Substantial space was thus given for the women's own stories and associations. Follow-up questions invited expansion of (Hydén, 2014) or elaboration on aspects of what the women had just shared. No interview questions were asked specifically about experiences of backlash. Sometimes, however, follow-up questions picked up threads that were categorized under this theme during the analysis.

The interviews were recorded and transcribed verbatim.

### Narrative Analysis

A basic premise of narrative analysis is that we make sense of our experiences through organizing them into, and conveying, meaningful episodes (Richardson, 1995). A narrative can be defined as a sequence of speech or writing through which events involving characters in a particular setting are connected and given meaning (Riessman, 2008). Experiences are thereby given coherence and significance, as meaning is constituted by the positioning of events in relation to each other (Richardson, 1995). Narratives about the self, the other, and the world develop in concert with such acts of interpretation, or of bringing “order out of memory” (Riessman, 2008, p. 23), which not only express but give form to meaning and identity (Kafka et al., 2019) within a social and historical context (Bruner, 1991). Narrative analysis, in its theoretical and methodological diversity, thus entails exploration of the participant's motivations or “symbolic system of orientation” (Hydén, 2014, p. 798), or of how events are assembled and language is used to communicate meaning (Riessman, 2008).

Regarding IPV, narrative analysis has pointed to public stories enabling some accounts to be more visible than others (Donovan & Hester, 2015). Narrative analysis has also shed light on how IPVAW is made sense of by survivors (Boonzaier & van Schalkwyk, 2011; Wood, 2001), perpetrators (Presser, 2009; Radcliffe et al., 2019), and professionals (Kafka et al., 2019). Such analyses have investigated how the causes of IPV were explained, through the attribution of causal relationships and meaning (Presser, 2009; Radcliffe et al., 2019). Along similar lines, this study looks at how IPVAW survivors ascribe causes to the violence through connecting events and formulating their meaning (Richardson, 1995).

The overarching theme of backlash was tentatively identified during the initial analysis of the interview transcripts. The transcripts were then re-read, and narrative segments relevant to the theme were coded as such. Segments were selected if they described violence as being provoked or driven by resistance to or challenging of the partner's control or position of superiority in the relationship. Such segments consisted of stories describing specific incidents, or habitual narratives referring to more general patterns (Riessman, 1990). Narrative segments describing the provocation of anger were included in the analysis, due to the continuous nature (Kelly, 1988) of anger and forms of IPVAW. Segments referring to jealousy were not included, although the line between jealous and controlling or violent behavior can be blurry. Segments about violence used to enforce the partner's control or position of superiority, but not as triggered by challenges to that position, were not included.

It should be noted that (quantitative) research on VAW has made important distinctions between relative and absolute measures of status; that is, between measures of gender equality (men's vs. women's status) and of socioeconomic position (women's status or men's status) (e.g., Roberts, 2011; Vieraitis et al., 2015; Xie et al., 2012). Meanwhile, studies incorporating the concept of backlash have used a range of measures including more absolute ones (e.g., Sabarwal et al., 2014). In this qualitative study, no distinction is made between relative and absolute status in the analysis of the women's narratives.

The focus of the study is the content rather than the form of the narratives, that is, the “told” rather than “the telling” (Riessman, 2008). The first part of the analysis was thematic. Following a process inspired by Braun and Clarke (2006), the contents of the narrative segments were grouped into categories (Table 1) using the software program NVivo. While this type of analysis pays little attention to the individual cases and settings, it can provide valuable information about narrative patterns (Riessman, 2008), not least regarding experiences related to power and subordination (Boonzaier & van Schalkwyk, 2011). In order to show divergences between how the identified themes appeared or did not appear in the participants’ narratives, the second part of the analysis was case-centered and consisted of the analysis of five individual interviews. While the thematic analysis is concerned with narrative segments, the case-centered analysis approached the interviews at large as narratives.

**Table 1.** Thematic Narrative Analysis: Example of the Process, From Data Extract to Theme.

Theme	Selected narrative segment	Subcategory	Category
Backlash	And I started studying. And if he noticed that I was studying I was put through hell sometimes … I didn’t dare to say [to my parents] that I went to school, in case they would tell him and he would flip out completely.	Education	Resources

The terms “story” and “narrative” are used interchangeably (Riessman, 2008). The interviews were held in Swedish, and the quotes have been translated into English by the author.

### Ethical Concerns

Due to the sensitive nature of the topic, and in consideration of recommendations for ethically sound research on IPV (Fontes, 2004; WHO & PATH, 2005), the interviewers strove to establish a calm and emphatic relational space (Hydén, 2014) during the interviews. Care was taken during communication prior to and subsequent to the interviews to minimize any risk of participation contributing to violence recidivism. A manuscript draft was sent to participants who had expressed an interest in taking part in writings before publication, for respondent validation enabling the correction of or discussion about interpretations (e.g., Frambach et al., 2013). No comments warranting manuscript edits were voiced by the responding participants. The study was approved by the Swedish Ethical Review Authority (Dnr 2019-00221).

## Results: Stories of Backlash

### Thematic Analysis

Stories of backlash, that is, narrative segments coded under this theme, were found in interviews with 20 of the 23 women. These segments were grouped into five categories concerned with personal experiences of what provoked the violence: the woman's *resources*, *agency*, breaking of *gender norms* and *resistance*, and *the partner's feeling of subordination*. An additional category encompassed stories referring to the *societal level* (Table 2).

**Table 2.** Thematic Narrative Analysis: Categories and Subcategories.

Themes	Categories	Subcategories
Backlash (*n* = 20)	Resources (*n* = 7)	Education (*n* = 6) Income (*n* = 3) Work (*n* = 2)
	Agency (*n* = 10)	Independence (*n* = 8) Freedom of movement (*n* = 4)
	Gender norms (*n* = 8)	Appearance and behavior (*n* = 3) Domestic work (*n* = 5) Threatened masculinity (*n* = 1)
	Resistance (*n* = 16)	Speaking back (*n* = 12) Resisting violence (*n* = 4) Drawing a limit (*n* = 3) Help-seeking (*n* = 2)
	Partner’s feeling of subordination/ disadvantage (*n* = 6)	
	Societal level (*n* = 7)	

#### Resources

Participants spoke about violence as being triggered by their existing or aspired resources, and a large share of this narrative content concerned *education.* Participants referred to meeting violent reactions when they expressed their will to get an education or progressively attained it, or when their academic training was referred to or acknowledged.

He didn’t think I should talk about it (…) because it bothered him terribly that I had an education, it bothered him terribly. (…) It was a power game (…) he had to push me down. (IP 4)

In relation to this, one woman referred to feeling “that I had to make myself smaller” (IP 13).

Some spoke about anger and violence being triggered by them having, or potentially having, a higher *income* than their partner.

[It] really deteriorated when I finished University and started working and making money, because then I made more money than him. (…) That made him crazy. (…) He would terrorize [me] because of that money. (…) This thing that the woman earns more than the man. That didn’t exist for him. It literally made him see red. (IP 20)

While several participants spoke about violent behavior obstructing their work and working conditions, two women referred to violence being triggered by their *employment*. One woman referred to an “acceleration” of violence (IP 21) when she fulfilled work demands, while another described being locked inside and thus physically hindered from going to work.

He didn’t want me to get any more education because he didn’t want me to be better off economically than him. And that was also why I couldn’t work. (…) He didn’t let me go there. He locked me inside on the same day that I was going to start. (…) And finally I couldn’t stay at that job because (…) I had too much absence. And that was … all because of the relationship with him, that he wouldn’t let me go to work. (IP 15)

#### Agency

While increasing restriction of the victim's agency and freedom of movement is a common feature of IPVAW (Lundgren, 2012) often noted in the interviews, this category includes segments describing violence as being provoked by victims expressing or claiming agency. Several of the participants referred to their *independence* as triggering violence.

If I was too independent and too strong in some way, then (…) then it was as if he had a need to kind of push me down. (IP 11)

Aspects of independence noted to have provoked violent responses included having opinions or interests, receiving attention from other people, maintaining any relationships other than that with the violent partner, or being happy.

When I talked about my job, then he could just scream. (…) If I spoke about my previous life (…) he became so enraged. (…) Children, work, my previous life, colleagues, anything (…) that didn’t involve him. So everything (…) All of that had to be cut off from my life. I was only supposed to be sure to satisfy him and his needs. (IP 13)

A few participants spoke about violence erupting when they claimed *freedom of movement*, through doing or wanting to do things such as seeing friends or parents, taking a swim, or leaving the house.

He wanted me to just be at home and fix things there, cook and stay there (…) Yes. So the more I tried to break loose and become my own, then he became … then everything got worse. (IP 17)

### Gender norms

Several participants spoke about violence being triggered by their deviations from traditional or desired gender norms, in ways other than those already discussed. Regarding *appearance and behavior*, some spoke about violence arising due to digressions from appropriate femininity, through, for example, being “too straight-forward” (IP 4) or not needing to be cared for.

It was important to him, this thing about masculine and feminine. And in his world there was a sharp border. And you couldn’t really walk across it really, but I did, I moved in both. (IP 4)

I’m not a woman you take care of, because I take care of myself. (…) He wanted a relationship with a woman he could take care of and be macho. (IP 23)

In this context, one woman spoke about tension between her partner's attitude towards gender norms in public situations and in the private sphere.

He spoke a whole lot outwardly about how proud he was that I have done military service and that I’ve worked as a security guard and that I have the position I have and that I make more money than him. But as soon as we got home, inside the doors, then it was … “Do you have to have short hair?,” “You look like a lesbian,” “Can’t you be a bit more feminine?” (IP 8)

Women spoke about violence being provoked by their partners thinking the women did not do sufficient *domestic work*, or by their asking or expecting the partners to contribute in this area.

A woman exists to be at and take care of the home—when the hell will you learn that? (IP 8)

If you don’t do it (…) then you just get beaten more or hear more that you’re worthless. (IP 5)

Relatedly, one woman spoke in more unspecific terms about her partner being triggered by a sense of having his *masculinity threatened*, due to destabilization of his superior status.

He has beaten me so many times. Because he was afraid. He was afraid that he would become a sissy and that I would be the woman with a higher position than him. (IP 9)

#### Resistance

Violence was described as provoked by resistance to the partner's will, authority, or violence, through the women's *speaking back*, *resisting violence*, *drawing a limit*, or *seeking help*.

Several women spoke about violence occurring as a result of their *speaking back* to the partner; of “not shutting up” (Int 4) or “question[ing] him” (IP 19). Keeping silent was also noted as a way of alleviating violence.

I answered back sometimes, I did. But then I knew it would get seven times worse. (IP 17)

The victim's speaking back was also indicated as the cause of violence attributed by a partner.

He admitted to the social services that he pushed me. (…) He said, “Well, but she knows what I’m like and still she wasn’t quiet.” (…) So I guess it was that … I mean, I felt that the violence came because I didn’t … I didn’t back off. I never became the silent woman. (IP 12)

Some women spoke about violence being provoked by or escalating due to them *resisting* (sexual/physical) *violence* more directly.

He forced himself on me sexually, he wanted to make me pregnant. But I went to the women’s clinic. (…) Then they gave me [contraceptive] patches. (…) He saw this, and (…) “You have a patch? Now you’ll see, you damn idiot.” And I tried to say, “But this is my body, this is my right … I decide.” No, it was impossible to talk. (…) And then he started beating me. (IP 22)

Some spoke about violence caused by them *drawing a limit* or setting a boundary. In the words of one woman, her partner became physically violent because “he couldn’t handle me putting my foot down … I mean, that I said that's enough” (IP 2). The relationship was gender equal in many ways, she added, as long as she did not resist his will.

As long as I yielded, it was fine. (IP 2)

In a couple of stories, violence was triggered by resistance in the form of *help-seeking* from the police or from neighbors.

#### The partner's feeling of subordination or disadvantage

Some narrative segments about the causes of violence included references to the partners’ perceived feelings of subordination or disadvantage, in relation to the victim, to women in general, or to the victim's family. One participant used the concept of an “inferiority complex” (IP 23), while other stories referred to low self-esteem overall, and to tension between such low self-esteem or a feeling of subordination and an image projected outwardly.

I think he … probably feels very subordinate to women. That this makes him have to, in some way, defend himself against that all the time, against the existence of women. (IP 10)

In one story, feelings of inferiority on the part of the partner were considered in terms of a possibility rather than an actuality, while the woman's similar feelings were noted to have influenced her perception of the violence.

It’s possible he didn’t feel he had as much power, and that’s why he had to use violence, I’m not sure. (…) My self-image was probably a bit strange, like I was super strong, like I was the strong one and he was weak, somehow. I guess that made me think, “I can handle this.” (IP 11)

#### Societal level

Some participants referred to backlash on the societal level, in most cases in response to an interview question about what they thought to be the cause of IPVAW on a general rather than a personal level. Here, participants sometimes referred specifically to Sweden.

In Sweden in particular, women have a lot of opportunities here. We can study, we can educate ourselves, we can have good jobs. And I think they see that as a threat, that women can be very independent here. So they need to have some way of controlling you, because they don’t know how to do it. And then it becomes through … physical violence. (IP 15)

In several cases, narrative segments including backlash on the societal level encompassed associations with the women's own personal experience. In this sense, the backlash theme represented a point of connection between a personal and a collective experience (Presser, 2009). In other cases, the segments separated personal experiences from the collective.

If you think you should be the person in charge in then … well, if the woman doesn’t want to submit to that, then I think there can be violence, because of that. But I think … in my case, the way I see it, there was something particular about [name]. (…) I know his father also hit. (IP 14)

This indicates critical reflection on backlash dynamics and a co-existence of perspectives on the causes of IPVAW.

### Case-Centered Analysis

While the noted categories reoccurred in the IPVAW narratives, the ways in which they appeared in the stories were quite different. The following section, therefore, consists of a closer look at the individual narratives of five participants.

#### Elise: “The cage”

Elise is a university-educated woman whose narrative contains all the noted personal-level categories. In this sense, her narrative is quite typical of or aligned with the overarching concept and theme of backlash as it appears in the interviews.

Elise told a story about her relationship with Patrik, both of whom were identified as Swedish. The relationship encompassed physical and psychological violence. At times it was relatively calm, however, provided that Elise “kept within the frames” by behaving in certain ways, “keeping calm” or being “this nice little girl” and letting Patrik be in a position of authority. Alongside the notion of frames, Elise repeatedly used the image of a cage when talking about the relationship and her adaptation to the violence.

As long as he could keep me in his little … then, then everything was fine. So it became, more and more, that I stayed in that little cage to … not upset him. (…) If I was inside that little bubble, we could live relatively normally, I think, in many ways.

Violence was described as being triggered by Elise's *resources*, as she spoke about Patrik being provoked by her studying and earning money. Violence was also associated with her *agency*, expressed through independent opinions and freedom of movement. In alignment with traditional *gender norms*, Elise referred to Patrik wanting her to stay at home.

I was supposed to be a classical woman, quite simply, this obedient little girl. […] Men should make money. Men should enjoy entertainments. Women should be at home and take care of the kids. […] He wanted these traditional roles. That’s what it was about … that’s what sums it up.

When asked about whether she had exerted any *resistance* toward Patrik, Elise responded that she had not dared to. She added that she sometimes spoke back to him, but that this caused the situation to deteriorate. Furthermore, she did not refer to her partner *feeling subordinate*, but connected his violence with low self-esteem and a related need to assert himself.

#### Anne: “He felt smaller”

Anne shared a story about her 25-year relationship with Markus. Holding a managerial position in a male-dominated workplace, Anne described herself as a strong and independent person, and she referred to herself and Markus as Swedish. Her narrative contained psychological and material violence, and physical violence from which she had visible scars. She related having stayed in the relationship due to fear of further violence including murder.

The categories of *resources*, *agency*, *gender norms,* or *resistance* were not present in the story. It did, however, feature a power relationship and the *partner's feeling of subordination*. The reason why Markus used violence, Anne said, was to reclaim a position of power.

That’s what I really think this is about, that he took back the power when he became violent. And that’s why I think that he must have thought I had it before. Because the only way to take power over me, that was by behaving in this way.

Anne spoke about having realized, despite her fear, that Markus felt smaller than or inferior to her, and that this was a reason for his violence. Markus's apparently non-violent relationship with a new woman, described as withdrawn and compliant, was used to support Anne's rendering of the violence as being due to Markus wanting to be in a position of authority. He had wanted, she said, to be the caring but dominant “big and strong bear.”

Instead I … I’m sure he has felt that I was … the big and strong bear in the relationship, which he couldn’t handle and wanted to pull down. That’s what I think happened.

This situation, she added, “created an inferiority complex in him,”

that he wasn’t man enough to take care of me, so then he felt smaller.

Thus, Anne's story places emphasis on the man's feeling of subordination, while illustrating that all the categories noted in the thematic analysis did not need to function as triggers of violence for backlash dynamics to be central to the IPVAW narrative.

#### Karoline: “I have to take care of this guy”

Karoline spoke about her 10-year relationship with Tobias, who subjected her to physical, psychological, and sexual violence. Karoline had a well-paid managerial job and referred to both herself and Tobias as Swedish.

Karoline spoke about violence as being triggered by her *resources*, including her job and her education which was higher than his, and by her *agency*, expressed through her speaking about other aspects of her life and maintaining social contacts. In terms of *resistance*, Karoline noted that violence was provoked or escalated when she spoke back to or questioned Tobias. Moreover, the story contained a sense of *subordination* on Tobias's part, but as perceived by her rather than him. A sense of being the stronger part in the relationship caused feelings that she needed to take care of and be extra sensitive towards Tobias.

I felt that I have to take care of this guy and be careful with him.

Karoline spoke about “toning down” her resources and agency to not make Tobias feel insecure, and increasingly limiting her own freedom and social interaction to prevent his jealousy, which made her feel sorry for him. Thus, she became “more and more compliant.”

Since I am perceived as so strong, maybe I scare him a bit. So I have to become even more kind and even more … like this. But (…) it just got worse and worse and worse.

Relatedly, Karoline noted that strong women may be particularly vulnerable to IPVAW.

I have my own theory that many strong women get in trouble, because they think precisely like this. (…) “No one can do this to me, because I’m too strong.” (…) We are too strong for our own good, because we tighten our fist in our pocket and just, “No, I will struggle a bit more.”

While this narrative contained many of the noted categories, then, it deviated somewhat from a story about an authoritative man lashing back due to loss of power, as Karoline saw herself as the stronger partner. This affected her acceptance of, and the development of, the violence.

#### Karin: “The kindest feminist guy ever”

Karin is a university-trained professional, whose relationship with Samuel had contained sexual, psychological, and physical violence causing her PTSD and remaining physical injuries. Karin identified Samuel and herself as Swedish. She called herself “a very strong and fairly independent person,” and Samuel “a prominent, eloquent feminist guy.”

Regarding *resistance*, Karin spoke of a consistent compromising of her boundaries. With reference to a form of “terror,” she described how Samuel not only did not respect her assertions of limits but met them with an intensified breaking of them. This pattern was particularly emphasized in the context of their sexual practice but was repeated in other spheres of life. In a rare instance of more assertive resistance at the time of their separation, Karin broke a material object as a way of “straightening myself,” after having “walked with my spine very subdued for such a very long time.” She spoke with regret of this act as she linked it to continued and repeated violence and control.

Concerning *agency*, Karin's narrative deviates from the more typical story as she was not socially isolated. On the contrary, she spoke of Samuel having encouraged her to engage with other people, but then using this as “an excuse for himself to then subject me to violence.” Regarding *gender norms*, Karin referred to Samuel's guilty conscience over any shortcomings in the area of a gender-equal division of domestic work as a driving force for his violence.

His bad conscience led to aggression. (…) So a whole lot was about him not getting a guilty conscience (…) cleaning in secret, and, like, pretending I hadn’t done anything (…) Because I knew that if I had (…) then there would be some kind of very strange form of punishment.

Relatedly, the driving forces for Samuel's violence were described as being interwoven with his identity as a feminist:

I know he felt bad about being a white upper middle-class man himself, in a way, and that he wanted to go out and defend all people … at the same time that he (…) With the pretext of going out to defend everyone he also violated me, because he also had to keep me weak, in a way.

When asked about what caused the violence, Karin pointed towards shame over the discrepancy between the way Samuel presented himself and the way he behaved,

the shame of getting turned on by hurting some for real (…) at the same time that you’re the kindest feminist guy ever.

Karin added that Samuel probably felt *subordinate* or inferior to women, and that this contributed to his violence.

Backlash dynamics are present in Karin's story, as her resistance to violent control and boundary crossings were met with more violence. The disjunction between Samuel's feminist principles and Karin's own experiences made it difficult for her to understand the violence and receive support, however. Meanwhile, Samuel's identification as a feminist was described as intermingled with guilt and shame, which became driving forces for further violence.

#### Sofie: “Punished for reporting”

Sofie, a university student, told her story about a relationship with Arif, which contained murder threats and psychological, sexual, economic, and material violence, as well as physical violence which started when she was pregnant. Sofie referred to herself as Swedish, and to Arif as having immigrated from a non-European country.

In Sofie's story, violence was sparked by her *resources*, including her job and her will to attain a higher education, and it escalated when she tried to practice *resistance* or did not fulfil *gender norms*. However, her narrative did not only concern Arif's violence, but was in large share focused on experiences subsequent to her disclosure of the violence to a midwife and then to the police. Due to concern for the newborn child, Sofie and her baby were taken into custody, where her mental health and parental ability were investigated.

It almost felt like I had been punished for reporting him to the police and telling the midwife about my problems. (…) It was really demeaning.

Sofie spoke about having been moved around “like a parcel” to different care homes, with her child, unable to make decisions about their situation and feeling criticized and blamed for the violence by care personnel. She was also facing the possibility of losing custody of her child.

That was my greatest fear, losing her. Because that’s what they threatened me with, repeatedly. (…) I was really really sad, really shocked by … how they worked, that they don’t have knowledge about intimate partner violence (…), how violence affects you and … how that can affect your parental ability temporarily. (…) It really felt like I was punished for making the report.

Sofie's story speaks not only of backlash dynamics in a violent relationship, then, but of feeling punished and threatened, and having her agency severely limited, by support institutions after resisting IPVAW through seeking help.

## Discussion

This study explored stories of backlash in interviews with 23 women exposed to IPVAW in Sweden, through thematic and case-centered narrative analysis. Narrative segments coded under the theme of backlash were found in 20 of the interviews. These referred to violence as being triggered by the woman's existing or claimed *resources* or independent *agency*, by her breaking with traditional *gender norms*, by her *resistance* to violence and control, or by the *partner's feeling of subordination*. In addition, some women's narratives pointed to backlash dynamics at the *societal level,* often associating these with the women's own experiences of IPVAW but sometimes separating them from personal causative factors. This indicates critical reflection about and a co-existence of perspectives on the causes of IPVAW.

These findings are in line with research pointing to backlash dynamics associated with economic or educational resources in Sweden and Norway (Bjelland, 2014; Ericsson, 2020). They also correspond with restriction of the victims’ agency and freedom of movement being a well-documented feature of violent relationships (Lundgren, 2012; Wiklund et al., 2010), and with resisting violence through leaving the relationship being tied to a heightened risk of lethal IPVAW (e.g., Ekbrand, 2006). Ample previous research has also pointed to the importance of norms related to gender and heterosexual coupledom.

Researchers have discussed how such norms can create relationship dynamics sliding towards and into IPVAW and its normalization in Sweden (Agevall, 2012; Brännvall, 2016; Edin et al., 2008; Gottzén, 2013; Wemrell et al., 2019; Wemrell et al., 2021). These include masculinity norms emphasizing virility and power, which may be conducive to violence, particularly when masculinity is perceived as threatened (Scheffer Lindgren & Renck, 2008a), and femininity norms emphasizing care, empathy and responsibility for, and adaptation to, others (e.g., Hydén, 1992; Lundgren, 2012). IPVAW has been described by perpetrators as being triggered by women abandoning femininity through being bossy, pushy, or making the partner “less of a man” by claiming power in the relationship (Edin & Nilsson, 2014), and the assumed acceptability of IPVAW has been posited as depending on the victim's femininity, on whether she was weaker than her partner or strong and thus “beatable” (Gottzén & Korkmaz, 2013). These findings point to ambiguous gender norms in contemporary Sweden (Hydén, 1992), where ideals of equality and mutual independence co-exist with more traditional ones, and where IPVAW may arise due to conflict between such changing (Flinck et al., 2005), co-existing (Fernbrant et al., 2013; Gottzén & Korkmaz, 2013), or confused (Edin et al., 2008; Wemrell et al., 2021) norms.

While the thematic analysis identified patterns in how the theme of backlash was expressed in the stories, the case-centered analysis pointed to diversities among them. Elise's story included all the thematic categories and was in that sense a more typical story, furthermore inviting cross-national comparison by sharing the use of the cage metaphor with Roy (2016), who writes about VAW and backlash in contemporary India.

While showing that all the noted categories need not be present for the backlash theme to be of relevance in an IPVAW narrative, Anne posited the partner's feeling of subordination as a central causative factor. This category was also important in Karoline's story, but with reference to Karoline's perception of herself as the stronger partner in the relationship. This self-perceived strength contributed to the unfolding of the violence. Relatedly, Donovan and Hester (2015) point to practices of love through which the abusive partner takes a position of emotional neediness making the victim emotionally responsible. This, they add, contributes to difficulties in recognizing the violence, as the dominant public story about IPV is about strong men violating weak women. While this was written primarily with reference to violence in LGBTQI relationships (Donovan & Hester, 2015), the point is also valid in relation to obstacles to identifying violence against women perceived as independent and strong.

Such difficulties have been observed by researchers noting that while IPVAW victims in Sweden often resist identifying themselves as such, particular resistance can be seen among women who are educated or self-identify as strong (Jarnkvist & Brännström, 2016; Scheffer Lindgren & Renck, 2008b). Relatedly, IPVAW victims with high socioeconomic status, or who show agency and confidence, have been less likely to receive legal support and redress (Agevall, 2012; Brännvall, 2016). This suggests that existing IPVAW support systems may be geared more towards women displaying passive, subordinate femininity, in alignment with notions of the “ideal victim” (Jarnkvist, 2015), than toward women claiming strength, agency, and equality, despite expectations of the latter for women in Sweden (Agevall, 2012; Brännvall, 2016). Wiklund et al. (2010) comment that in Sweden women tend to be dichotomized as being either strong or potential IPVAW victims. While actualizing the ambiguity of femininity norms in Sweden (Hydén, 1992), this is noteworthy with regard to backlash dynamics, which are likely to affect women perceived as resourceful and strong. Relatedly, women with relatively high education or income are often overlooked in discussions of how to address IPVAW in Sweden (Ericsson, 2020), as these are often focused on particularly vulnerable groups. While attending to the needs of marginalized groups is certainly warranted, this orientation bears the risk of obscuring the relevance of societal power structures for IPVAW in Sweden and contributing to tendencies towards the Othering of IPVAW, by positing it as located in particular—notably, immigrant—groups (e.g., Wemrell et al., 2019).

Karin's narrative, even more so than Karoline's, shows that IPVAW perpetrators and relationships do not all look the same, and that the presence of feminism does not necessarily safeguard against the exercise of power and IPVAW. In Karin's story, the discord between the partner's feminist identity and his violent actions blended with guilt and shame, which became driving forces for further violence. Corresponding references to discrepancy between IPVAW perpetrators’ principles and actions (Berggren et al., 2020; Boethius, 2015), and to associated shame (Gottzén, 2016), can also be found in previous research from a Swedish context. Such discrepancy, which in Karin's story made it hard to discern and understand the violence, is reminiscent of references to the co-existence of gender equality and IPVAW in Sweden as indicating a gap between principle and practice (Gottzén, 2014), between ideal and reality (Stubberud et al., 2018), or between a gender-equal surface level and misogynistic dynamics playing out below (Wemrell et al., 2021). Meanwhile, this discord invokes the question of how men endorsing gender equality (Gottzén, 2013) can expose their partners to violence.

Studies of how IPVAW perpetrators in Sweden make sense of their violence have identified patterns of blaming particular circumstances, including the victim or something other than the real self, such as alcohol, neurochemistry, or loss of control (Boethius, 2015; Edin & Nilsson, 2014; Gottzén & Korkmaz, 2013; Håland et al., 2016). Such patterns include the redefinition of violence, often signaling its mutuality, and, including after prosecution or help-seeking, disidentification with IPVAW perpetration (Boethius, 2015; Edin & Nilsson, 2014; Gottzén & Korkmaz, 2013). Researchers have here referred to cognitive dissonance (Håland et al., 2016), forgetting or repressing violence (Gottzén & Jonsson, 2012), or an absence of the self as an agential subject (Hydén, 1992) in perpetrators’ accounts of IPVAW. IPVAW perpetrators in Sweden, Gottzén and Jonsson write (2012, p. 150), “try to avoid the shadowlike figure that is pointed out as the Other of gender equality, while not necessarily trying to become gender equal men”. Through patterns such as these, then, IPVAW can apparently co-exist with the stated rejection (Karlsson et al., 2020) of its acceptability.

Sofie's story spoke not only of backlash dynamics in her violent relationship, but also of feeling punished or threatened by societal organizations after having reported the violence. This corresponds with research pointing to some IPVAW victims’ experiences of revictimization through contact with legal, healthcare, or social service organizations in Sweden (Agevall, 2012; Brännvall, 2016; Häggblom & Möller, 2007; Pratt-Eriksson et al., 2014; Örmon & Hörberg, 2016; Ulmestig & Panican, 2015), or of such institutions being felt to do the perpetrator's bidding (Bruno, 2018; Eriksson & Ulmestig, 2021; Jarnkvist, 2015; Jarnkvist & Brännström, 2016). Such experiences are associated with tendencies towards victim-blaming (Bengtsson-Tops et al., 2009; Pratt-Eriksson et al., 2014; Örmon et al., 2014), which are in turn linked to individual-oriented ways of understanding IPVAW and related assumptions of existing gender equality (Ekström, 2018; Hoppstadius, 2018; Mattsson, 2011). The notion of surface-level gender equality, noted above, has also been related to IPVAW support often being insufficient in practice (Wemrell et al., 2021). Such a gap between laws or policies and their implementation in Sweden have been pointed out repeatedly (GREVIO, 2019; Kvinnolobby, 2021; Wemrell et al., 2020).

Furthermore, one of the categories noted in the thematic analysis was the *partner's feeling of subordination* or disadvantage, as perceived by the women. That is in line with feelings of powerlessness long having been posited as an important factor behind men's violence against women in Sweden (Isdal, 2001). This underscores the importance of men's mental health, which is insufficiently understood and treated (Danielsson, 2010), likely due in part to masculinity norms (Danielsson, 2010; Krumm et al., 2017), despite its correlation with aggression and violence (Krumm et al., 2017; Yu et al., 2019). This category is also of relevance in relation to notions of male victimhood used in anti-feminist mobilizations (Chouliaraki, 2020), for example, in international incel communities where women and feminism are blamed for some men's inability to form intimate relationships (Gotell & Dutton, 2016). Such incel milieus, in which Swedish men have a strong presence (Fernquist et al., 2020), have been tied to VAW (Hoffman et al., 2020). Moreover, this category can be related to recent research showing that a large share of, particularly young, men in Sweden report blaming feminism for some men's feelings of societal marginalization (Mulhall & Khan-Ruf, 2021).

The latter, in turn, can be seen as expressive of a general backlash against gender equality in Sweden and elsewhere (EU, 2018), and with decreasing support for gender equality among men in nations with higher country-level gender equality, which Kosakowska-Berezecka et al. (2020) suggest may help explain the Nordic Paradox. Correspondingly, young men in Sweden express gender-conservative values and the opinion that women exaggerate levels of gender inequality (Swedish Gender Equality Agency, 2021). IPVAW professionals in Sweden, meanwhile, point to signs of backlash in younger generations, in the forms of lenience toward more traditional gender roles and an acceptance of IPVAW (Wemrell et al., 2021).

### Other Stories: Narrative Content Falling Outside of the Theme

All the participants’ stories about violence causation, or about agency or resistance, did not fall within the theme of backlash, which is the focus of this article. In a couple of cases, narrative segments about acts of resistance did not speak about backlash, that is, about the provocation of further violence, but about the ending of the relationship. Thus, when the women “started answering back” (IP 14) or “showing some attitude, instead of being subordinate” (IP 19), the partner left. In one interview narrative, which was one of the few containing no content coded under backlash, the woman noted that the relationship provided “no space” (IP 3) for resisting violence. In another, the woman spoke about hardly having considered resisting violence or making claims for gender equality, as her partner had the power “one hundred percent all the time” (IP 7). Thus, these narratives were less about backlash and more about violent partners’ authority remaining unchallenged.

When speaking about causes of violence, only one narrative referred to speaking back to or questioning the partner's authority as the sole trigger. All other stories contained other causative factors. Many spoke of jealousy or dynamics of power and control, not coded under the theme of backlash as there was no mention of violence being triggered by resistance to that power or control. Aside from this, and in line with previous research showing a co-existence of structural and individual-level explanatory factors in IPVAW victims' accounts (Agevall, 2012; Brännvall, 2016; Gottzén & Korkmaz, 2013; Krumm et al., 2010b), many spoke of the perpetrator's mental ill-health, perceived pathology, or substance use. Several referred to the perpetrator or victim's previous experience of violence, or to aspects of relational patterns or background factors, for example, being adopted or lacking parental attachment. Racism was also mentioned as a factor. Backlash dynamics thereby appeared as one cause among others.

Alongside references to other causative factors, and also in accordance with previous findings (Agevall, 2012; Brännvall, 2016; Enander, 2010a; Hydén, 2005; Örmon et al., 2014), several narratives contained elements of victim-blaming. Many women described having been blamed by their violent partners, by themselves, or, in a few cases, by other persons or societal institutions, for the IPVAW. Several spoke about a process of realization that the violence was not their fault. While victim-blaming is a cross-national phenomenon, self-blame and associated feelings of shame, guilt, and stupidity for having “allowed” or “subjected oneself to” IPVAW in the context of assumed gender equality have been pointed out among IPVAW victims in Sweden (Brännvall, 2016; Edin, 2006; Enander, 2010a; Hydén, 2005). Such notions may form obstacles to help-seeking and to leaving violent relationships (Enander, 2010b).

This presence of both victim-blaming and backlash in the narratives is noteworthy as these themes do quite different things, in terms of constructing identities and encouraging actions (Riessman, 2008). With reference to Richardson (1995), Presser (2009) distinguishes between “cultural stories,” told from the point of view of the ruling or normative order and contributing toward maintaining the status quo, and “collective stories,” which can assist social change by giving voice to those silenced or marginalized in cultural narratives, including IPVAW survivors. While victim-blaming, associated with an individual-level explanatory model for IPVAW, can be seen as a “cultural story,” “collective stories” about backlash may enable emancipatory practices through the grasping of dynamics of power.

### Limitations

The act of narration in an interview situation is an active sense-making process, involving the narrator and the interviewer (Hydén, 2014), as well as the societal context (Riessman, 2008), and the presence of any cultural or collective stories (Presser, 2009) or scripts (Bletzer & Koss, 2004). Accordingly, while many participants emphasized that they had done a lot of thinking and processing of the experienced violence, some noted that they remembered or connected things during the interview. It is worth noting here that the concept of the Nordic Paradox, and the project's interest in gender (in)equality, was outlined in the invitation letter to potential participants, and in the information about the project given at the beginning of the interviews. This may have had some effect on the contents of the narratives, and it is possible that women with an interest in links between IPVAW and gender equality were more likely to volunteer to take part. That said, no interview questions inquired about backlash effects. The narratives unfolded in response to open and broad questions, as the interviewers strove to give extensive space for the participants’ own stories and balance their own involvement (Hydén, 2014) in the narratives to avoid imposing concepts or interpretations upon them.

By focusing on IPVAW, this study can be said to align with a dominant public story about IPV (Donovan & Hester, 2015) as something that only affects cis-gender females. As all participants’ stories were about heterosexual relationships, this study also disregards violence in LGBTQI relationships. This very important topic has been (Donovan & Hester, 2015; Holmberg et al., 2005) and is researched elsewhere.

While the socioeconomic status of the participants differed, it is possible that they were more socioeconomically privileged than IPVAW victims in Sweden overall. This could mean that they were more likely to experience violence due to backlash dynamics (Ericsson, 2020).

Finally, this qualitative study cannot shed light on the extent to which the high levels of IPVAW in Sweden (FRA, 2014) can be explained by backlash dynamics. It does, however, provide information about the stories of backlash which appeared, unprompted, in many of the narratives of the interviewed IPVAW survivors.

## Conclusion

In line with the amelioration hypothesis, high IPVAW prevalence rates in Sweden and neighboring Nordic countries (FRA, 2014) have been posited as being paradoxical (Gracia & Merlo). Correspondingly, research has pointed to tendencies towards seeing IPVAW perpetration as the “the Other of gender equality” and, as such, the Other of the Swedish man (Gottzén & Jonsson, 2012, p. 150). Understanding the apparent puzzle of IPVAW in Sweden has been pointed out accordingly as being of importance to researchers, policy-makers, and practitioners in both international and regional settings (Eriksson & Pringle, 2005).

Meanwhile, in international research on VAW, the amelioration hypothesis has long been accompanied by the backlash hypothesis—a co-existence that has also been termed paradoxical (Whaley et al., 2013). From the perspective of the backlash hypothesis, according to which women's empowerment may trigger violent resistance or retaliation, high IPVAW prevalence rates in Sweden and other Nordic countries are perhaps not so paradoxical after all. While this qualitative study cannot measure the importance of such backlash dynamics in relation to other causative factors for IPVAW prevalence in Sweden, it indicates their potential relevance. Backlash dynamics, in various ways, formed part of most of the IPVAW narratives told by the interviewed women. This underscores the complexity of gender equality and its associations with IPVAW (Latzman et al., 2019). It also suggests that women's progressive empowerment may not only shift from being a risk factor for IPVAW to being a protective factor (Schuler & Nazneen, 2018; Whaley et al., 2013), but also may invoke further backlash dynamics in the form of violence (Håkansson, 2019; Stamatel, 2018).

While a combination of causal factors is surely of relevance to IPVAW (Heise, 1998), focusing on individual-oriented explanations may render backlash and other power dynamics invisible. This may create, in turn, additional obstacles to the identification, addressing, and prevention of IPVAW, and potentially affect violence recidivism and prevalence. Relatedly, while gender-neutral approaches are important for moving beyond reductionist assumptions of perpetrators and victims, and associated limitations in the prevention and addressing of IPV, they should not obscure the relevance of power dynamics. This is pertinent not least in the context of contemporary signs of a backlash against efforts towards gender equality in Sweden and elsewhere (EU, 2018; Kosakowska-Berezecka et al., 2020; Mulhall & Khan-Ruf, 2021).

In conclusion, this study suggests that IPVAW research, policy, and practice should remain attentive to the potentially complex impacts of gender-related power dynamics on IPVAW causation, and to their importance for addressing and preventing IPVAW in Sweden and elsewhere.
